# Special Issue “Molecular and Cellular Advances in Atopic Diseases”

**DOI:** 10.3390/ijms25094856

**Published:** 2024-04-29

**Authors:** Beatriz Cabanillas

**Affiliations:** Department of Allergy, Instituto de Investigacion Biosanitaria Hospital 12 de Octubre (imas12), Avenida de Cordoba s/n, 28041 Madrid, Spain; bcabanillas.imas12@h12o.es; Tel.: +34-913908763

Atopic diseases, which currently affect around one billion people worldwide, are experiencing a rising prevalence [1,2]. Atopic diseases are characterized by intricate pathophysiological processes that are shaped by several molecular and cellular factors. Recent progress in the elucidation of the mechanisms of these diseases has enhanced our knowledge of their pathophysiology, sensitization pathways, and the roles played by specific immune cells in their onset, progression, and management. This Special Issue, entitled “Molecular and Cellular Advances in Atopic Diseases”, is dedicated to exploring new insights into the molecular and cellular foundations of atopic diseases. It also seeks to present innovative findings related to the regulation, treatment, diagnosis, and prevention of these conditions.

Allergic sensitization involves two crucial initial stages: first, the release of allergens from their natural sources; and second, the interaction of these allergens with human epithelial barriers. The release of allergens from their natural sources has been extensively analyzed in various pollens, such as those from birch and olive tree [3,4]. Different studies have demonstrated that hydration significantly enhances the release of pollen allergens, particularly major allergens over minor allergens. For instance, major allergens Bet v 1 from birch and Ole e 1 from olive tree are more readily released upon hydration compared to their minor counterparts [5,6,7]. This rapid release pattern of major allergens contributes to their allergenic potential due to a faster and higher interaction with epithelial surfaces [8,9]. During thunderstorms, pollen grains absorb water, which increases their hydration and leads to the release of allergenically active sub-pollen particles. These particles can penetrate the airways of sensitized subjects, and induce thunderstorm-related asthmatic symptoms [10,11,12]. Subtropical weather in areas such as the Canary Islands has been found to increase permanent exposure to house dust mites (HDM) and storage mites with a high prevalence of IgE sensitization to major HDM allergens in contrast to minor allergens [13]. These studies highlight the distinction between major and minor allergens: major allergens, known for their higher immunogenicity and prevalence, tend to be released more readily from their biological sources compared to minor allergens. The propensity of major allergens for rapid release enhances their potential to trigger allergic responses in susceptible individuals [5,14,15].

Regarding food allergens, the dynamics are somewhat different but equally significant. In studies involving peanuts, it was found that major allergens Ara h 1, Ara h 2, and Ara h 3 are rapidly released into saliva during the act of mastication, with Ara h 1 showing increased levels in saliva the longer the mastication process lasts [16]. Allergic reactions to peanuts by inadvertent exposure through saliva have been described in the scientific literature. Data from different studies and case series demonstrated that severe reactions to peanuts after kissing individuals who have eaten peanuts in the previous hours are not rare [17,18,19,20,21,22]. In that respect, it is known that during a kiss, 88 µg of peanut protein can be transferred through saliva [23], and the reactions may occur even after regular oral cleaning with toothbrush, mouth rinsing, and gum chewing upon peanut consumption [18,19]. This accidental exposure to peanuts constitutes a source of concern for peanut-allergic patients. In that sense, a recent study demonstrated that Ara h 1, unlike Ara h 2, strongly adhered to the oral epithelium even after oral cleansing, showing high persistence in the oral epithelium. This strong persistence of Ara h 1 in the oral area may explain the cases of allergic reactions after kissing individuals who were previously exposed to peanuts [16]. Recently, it was demonstrated that when peanut seeds are hydrated, major peanut allergen Ara h 1 was quickly released, reducing its content in the seed, while Ara h 2 remained relatively preserved. Ara h 3 showed no decrease in content, despite its release into the hydration water. Minor allergens Ara h 8 and Ara h 9, which are less abundant in peanuts, also transferred into the water, leading to a reduction in their seed levels [24]. Specific heating conditions, such as boiling, have also been found to facilitate the release of low molecular weight allergens such as Ara h 2 from peanuts into the boiling water, highlighting how thermal processing affects allergen dispersion [25,26]. These studies collectively highlight that the release kinetics of allergens from their natural sources, the differential characteristics between major and minor allergens, and their interactions and persistence within human epithelial barriers are key factors in the sensitization phase of allergy.

The interaction of allergens with the epithelial barriers can be influenced by several factors that may directly or indirectly affect the integrity of the epithelial barrier. In that respect, the epithelial barrier hypothesis was recently described as a concept proposing that environmental changes related to urbanization, industrialization, and modernization disrupt and inflame human epithelial barriers [27,28,29,30,31]. Damage to the epithelial barriers by these factors was suggested to facilitate the entry of allergens and pathogens, disrupting local immune responses, and promoting type 2 inflammation, inducing the initiation and exacerbation of allergic diseases such as atopic dermatitis (AD), food allergy, and asthma. The hypothesis also highlights that structural and functional changes in the epithelium induced by environmental insults could lead to changes and loss of microbial diversity, a condition known as dysbiosis, which is associated with a range of chronic inflammatory diseases [27,32] (Figure 1).

In the context of the epithelial barrier hypothesis, recent studies have demonstrated that certain food additives, such as artificial emulsifiers, disrupt the integrity of the intestinal epithelial barrier, inducing an inflammatory response [33]. Additionally, exposure to chemical residues in utensils exposed to dishwasher rinses has been shown to alter the expression of genes related to cell survival of the epithelial barrier, cytokine signaling, and metabolism, causing inflammation and damage to the intestinal epithelium [34].

The epithelial barrier hypothesis has had such an influence that a new updated nomenclature of allergic diseases and hypersensitivity reactions has included it within the broader categorization of hypersensitivity reactions. This modern nomenclature approach acknowledged the role of epithelial barrier defects in driving immune dysregulation and hypersensitivity, classifying these mechanisms under the Type V hypersensitivity category. This classification reflects a deeper understanding that disruptions to epithelial barriers, induced by various factors, play a pivotal role in the pathogenesis of allergic diseases [35].

Environmental and genetic factors contribute to the pathogenesis of diseases closely linked to defects in the epithelial barriers, such as AD. In that respect, multiple defects in AD skin have been characterized, such as (loss-of-function) mutations in the FLG gene, which encodes filaggrin, a key protein in maintaining skin barrier integrity, and in the TMEM79 gene, which affects the secretion of specific proteins key in stratum corneum formation, and in the SPINK5 gene, which is involved in skin barrier formation and inflammation regulation. These genetic variations significantly impact the structure and function of the skin, exacerbating the AD condition [36,37,38,39,40]. Inflammation and alterations in skin metabolic pathways also contribute to the symptoms of AD. In that respect, an altered NAD+ and PAR metabolism, which play a critical role in regulating cell survival and function, have been found in the skin of AD patients, showing a strong correlation between lesional AD status and NAMPT and PARP1 expression [41]. Moreover, the NLR family pyrin domain containing 3 (NLRP3) inflammasome is a critical element of the innate immune system, playing a key role in inflammation. Its altered expression has been linked to the exacerbation of AD, making it an important target for therapeutic intervention, which is an aspect addressed in one of the articles of this Special Issue [42].

Other studies have shown that viral pathogens, such as Herpes Simplex Virus type 1 (HSV-1), significantly contribute to the exacerbation of AD, particularly in conditions such as AD complicated with eczema herpeticum (ADEH). In that respect, it has been found that ADEH patients have significantly higher levels of specific IgE against HSV-1 compared to both AD patients and healthy controls. This suggests that HSV-1 may contribute to the severity of AD through IgE-mediated pathways, potentially exacerbating symptoms through immune dysregulation that favors a Th2-skewed response, which is ineffective against viral pathogens but proactive in allergic inflammation [43,44]. In line with this assumption, it was also found that specific viral proteins, such as glycoprotein D from the HSV-1 envelope, significantly increased the infiltration of inflammatory T cells and mast cells into the skin of a mouse model of AD, thereby promoting inflammation similar to that observed in human AD. These findings underscore the potential of viral components to act as allergens themselves, further altering the skin immune response in AD [45].

Another critical factor in contemporary daily life that can significantly affect the health of epithelial barriers, and thereby contribute to disease development, is the increased consumption of diets rich in ultra-processed foods, including processed meats [46,47]. To counteract this high consumption, healthier dietary options are being introduced to the market. Legumes, for instance, are seen as an excellent alternative to meat protein, and their regular consumption is linked to a lower risk of cardiovascular diseases within a healthy lifestyle framework [48,49,50]. Furthermore, with the growing interest in gluten-free diets, foods made from legumes have increased their presence in the market, such as alimentary pasta made of legumes [51,52]. However, legumes can cause severe allergic reactions; therefore, the introduction of novel foods and new ingredients into the market carries potential risks for individuals with food allergy. In that sense, severe reactions have been reported in patients allergic to legumes when consuming pasta fortified with lupine [53,54,55]. Alimentary pasta made entirely from legumes is presented in formats very different from the legume seed (such as in the form of spaghetti, macaroni, fusilli, etc.), which may confuse and pose risks to those individuals with legume allergy. For that reason, recent studies have conducted thorough analyses to determine the allergenic potential of new forms of legume-based pasta, and to study how food processing used to consume these products might alter their allergenicity. In that respect, it was found that marketed alimentary pasta made of 100% chickpeas retains a high content of allergenic proteins, similar to that found in hydrated and boiled chickpea seeds. The allergens identified included 7S globulin, 2S albumin, LTP, and PR-10. The study also demonstrated that the process of boiling transferred more allergens from the alimentary chickpea pasta to the boiling water compared to chickpea seeds. This finding suggests that while boiling reduces allergen content in the solid food, it potentially increases the allergenicity of the boiling water, presenting a risk to sensitized individuals [56]. Another study showed that alimentary pasta made of 100% lentils has a significant allergenic content that is closely similar to that of the original lentil seeds. Both the pasta and the seeds displayed a substantial transfer of allergenic proteins to their respective boiling water during boiling processing. This similarity in allergenic profiles between the pasta and the seeds indicates that the novel pasta format contains an important allergenic potential [57]. These findings highlight the need for careful assessment of allergenic risks when introducing new plant-based food products into the market, especially those aimed at consumers with dietary restrictions related to food allergy. In this context, it is also important to investigate how food processing can alter the allergenicity of foods, as it can increase, decrease, or have a neutral effect on it [58]. Moreover, food processing not only has the potential to alter the IgE recognition of food proteins, but also to change the recognition and processing of these foods by dendritic cells in the context of the sensitization phase of food allergy [59].

Nuts, tree nuts, cereals, and seeds constitute other relevant sources of plant protein in vegan diets. In this context, it is crucial to not only continue deepening the characterization and identification of food allergens from these biological sources, but also to thoroughly characterize their IgE-binding epitopes at a molecular level [60,61,62,63,64]. Understanding the molecular properties of plant-derived food allergens and their epitopes will not only enhance the analysis of new plant-based foods, but is also a vital step toward the diagnosis of food allergy using individual components, a method referred to as component-resolved diagnosis.

## Figures and Tables

**Figure 1:**
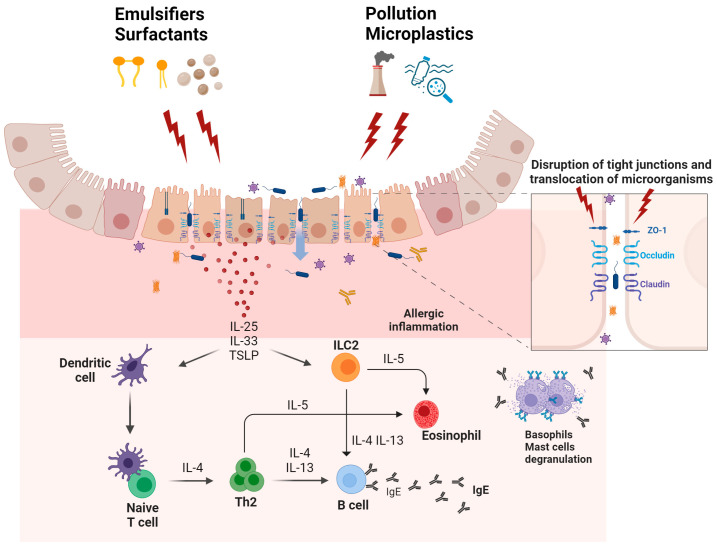
The cellular and molecular bases of the epithelial barrier hypothesis in relation to allergic inflammation. In response to environmental stimuli, such as artificial emulsifiers, detergents, pollution, etc., epithelial cells release cytokines, such as IL-25, IL-33, and thymic stromal lymphopoietin (TSLP), which shift immune responses towards a Th2 dominance. IL-25, IL-33, and TSLP induce the activation of dendritic cells and group 2 innate lymphoid cells (ILC2s). ILC2s contribute to the production of Th2-like cytokines including IL-5 and IL-13, amplifying allergic inflammatory responses. This cascade also recruits eosinophils, basophils, and mast cells to the affected area, leading to degranulation and further inflammation. Additionally, disruption in the epithelial barrier through tight junctions’ disruption can cause increased permeability, altering the microbiome balance—reducing beneficial commensals and increasing opportunistic pathogens. This microbial imbalance promotes the migration of microorganisms into epithelial and subepithelial layers, exacerbating inflammation. Biorender software (version 2024) was used to create this figure under an academic license.
